# Development of a neurocognitive test battery for HIV-associated neurocognitive disorder (HAND) screening: suggested solutions for resource-limited clinical settings

**DOI:** 10.1186/s12981-019-0224-4

**Published:** 2019-04-15

**Authors:** Lai Gwen Chan, Mei Jing Ho, Yijun Carol Lin, Yining Ong, Chen Seong Wong

**Affiliations:** 1 Department of Psychological Medicine, Tan Tock Seng Hospital, Annex 1 Building Level 3, 11 Jalan Tan Tock Seng, Singapore, 308433 Singapore; 2 National Centre for Infectious Diseases, Tan Tock Seng Hospital, 16 Jalan Tan Tock Seng, Singapore, 308442 Singapore

**Keywords:** HIV-associated neurocognitive disorders, Cognitive impairment, Screening, Neuropsychology

## Abstract

**Background:**

Practical screening strategies are necessary to detect neurocognitive impairment of all severities in HIV populations, which remains prevalent despite highly active antiretroviral therapy and requires full neuropsychological testing for diagnosis. We aimed to develop a brief and clinically feasible battery to screen for HIV-associated neurocognitive disorders (HAND) in resource-limited settings even where English is not the native language.

**Methods:**

A total of 53 outpatients were recruited from a multi-ethnic Southeast Asian HIV-positive cohort. Performance on a neuropsychological protocol was used to define cognitive impairment, of which 28 patients (52.8%) were identified with HAND. Receiver operating characteristic analysis was used to determine the best combinations of cognitive tests for the screening battery.

**Results:**

3 different combinations of cognitive tests that required minimal literacy, time to administer, and administrator training were found to classify HAND with fair accuracy. Montreal Cognitive Assessment (MoCA), in combination with tests of psychomotor coordination, verbal learning and speed processing, yielded area under curve scores of above 0.75, the primary outcome of receiver operating characteristic analysis.

**Conclusion:**

The 3-test combinations presented in this study appear to be promising screening options for HAND in HIV-infected patients. The addition of 2 tests to MoCA improves the overall accuracy while retaining its convenience, giving more potential for the inclusion of cognitive screening in routine clinical care. Further validation of the batteries in specific settings is warranted to determine specific screening cut-offs to a global cognitive score.

## Introduction

Despite the introduction of highly active antiretroviral therapy (HAART), the prevalence of HIV-associated neurocognitive disorders (HAND) remains high; up to 50% of HIV-positive individuals are estimated to experience some level of neurocognitive impairment [1]. It was observed that approximately 1 in 5 HIV-positive patients from a South Asian population can be expected to be diagnosed with HAND [2]. HAND has become a chronic end-organ complication impacting quality-of-life and functional outcomes, now that survival outcomes of HIV have improved greatly with the advent of HAART [1, 3–5]. Even in its mild form, HAND is independently predictive of death [6] as well as HIV-associated dementia [7]. Consequently, understanding HAND is essential in the long-term clinical management of HIV and AIDS together with the daily functioning of HIV-positive individuals.

Over the years, diagnostic criteria for HAND have evolved to better reflect the changes in the understanding of the pathology and epidemiology of HIV, with the Frascati criteria being the most recent [8]. The current nosology for HAND covers three disorders ranging in severity from asymptomatic neurocognitive impairment (ANI) to mild neurocognitive disorder (MND) to HIV-associated dementia (HAD). Cognitive performance and report of functional impairment form the basis for the diagnostic criteria of HAND, with functional decline in MND and HAD patients and ANI patients lacking functional complaints. The recommended assessment for HAND involves a comprehensive neuropsychological protocol that encompasses multiple domains by using standardized tests with published normative data [8, 9]. To our knowledge, no validated screening tool is yet capable of discriminating between levels of cognitive impairment associated with varying HAND severities.

However, as completion of the entire protocol is often cumbersome and time-consuming, there is a rising demand for brief, streamlined neurocognitive assessment tools that guide the preliminary identification of patients who require further testing. Several cognitive assessment tools such as Mini Mental Status Exam (MMSE) and International HIV Dementia Scale (IHDS) are commonly employed to detect HIV-related neurocognitive impairment in time and human resource-limited settings [8, 10–12]. These tools were largely developed for identifying dementia symptoms and therefore predominantly target cognitive functions (e.g., naming errors, visuospatial deficits) that stem from posterior neocortical deficits [13]. HAND, however, is typically milder in degree of impairment and more often impacts cognitive functions accounted for by the subcortical frontostriatal regions (e.g., processing speed). In particular, Morgan et al. [14] reported IHDS as only 50% sensitive in detecting asymptomatic neurocognitive impairment even after demographically adjusting the cut-off. These cognitive screeners, when used alone, often possess an unsatisfactory sensitivity and reliability in detecting mild neurocognitive impairment in HIV populations [15, 16]. No single tool was proven to be ideal in screening for symptomatic HAND in a 2016 study by Joska et al. [17] to which the authors proposed using additional tests or combined screeners for increased accuracy. The availability of a screening paradigm that can easily be administered to predict HAND is therefore necessary.

Despite the aforementioned inadequate reliability when used singularly, these tools measure several cognitive domains simultaneously and are fairly quick to administer. In addition, they are easy to comprehend and require only minimal training and material demands, rendering them an excellent choice in a brief cognitive test battery. A major concern with utilising such a screener is that majority of them was validated in North America with recommended cut-off values that were based on demographic characteristics of said samples. The scarcity of validated assessment tools with normative data in resource-limited and non-English speaking settings constitutes an additional obstacle to the detection of and intervention for HAND. The possibility of inaccuracy in screening HAND is larger when the specific demographic differences are not corrected for [18]. Unreliable diagnosis of HAND can lead to inappropriate medical intervention which then has profound negative implications on self-esteem and future financial and life planning [19]. A comparison between the commonly used screeners found that MMSE displayed poor sensitivity and is not recommended for diagnosing HAND [15, 17, 20]. When evaluated in HIV patients in the United States, Montreal Cognitive Assessment (MoCA) appeared to fare slightly better than IHDS, producing sensitivity of 90% and specificity of 87%, [21] as compared to the latter’s 88% sensitivity and 57% specificity [10]. IHDS was found to be poor in detecting HAND in 75 HIV-positive Thai patients, while the addition of Trail Making Test A was most effective in improving its sensitivity and specificity [22].

In light of the challenges, the current study aimed to develop a brief cognitive screening battery that produces the highest possible sensitivity and specificity in screening for HAND, for use in routine clinical practice in non-English speaking populations and resource-limited settings where full neuropsychological testing is not readily available, even in high-income countries like Singapore. While there have been previous attempts at assembling abbreviated screening HAND procedures, most are not readily adaptable or validated for other cultural settings [13, 15, 18]. Hence this study is designed to be pilot and exploratory, with the intention to further refine and validate the recommended screening battery in future. The authors’ approach was to identify commonly used neurocognitive tools whose combination requires minimal resources to administer yet yields acceptable classification accuracy rates. The tests included in the brief battery were selected using a theoretical framework that reflects the cognitive domains thought to be frequently affected by HAND [23].

## Methods

This study was conducted between March 2013 and March 2014 in Tan Tock Seng Hospital, Singapore. A total of 53 HIV-infected patients were recruited from the Communicable Disease Centre (CDC), which is Singapore’s largest referral centre for care and treatment of HIV/AIDS. Patients were eligible if they were HIV-positive, aged above 21 years old and were able to communicate in English. Also, all eligible participants were required to score 7 or above on the Abbreviated Mental Test (AMT) and were deemed by their primary Infectious Diseases physician to be medically well for the study. Patients who declined to participate, were not verbally communicative, or had scored > 10 (moderate) on the Hospital Anxiety and Depression Scale were excluded from the study. These criteria were used so as to be broadly inclusive, yet excluding those with clinically significant and active neurological and psychiatric conditions that could confound the results. All study participants provided written informed consent and the study was approved by the Domain Specific Review Board of the National Healthcare Group.

Basic demographic data such as age, gender, race and educational level were collected from all participants. Subsequently, the participants were required to complete a comprehensive neuropsychological protocol that comprised 12 cognitive tests. Emphasis was placed on ease of test administration as the objective was to develop a battery that could be easily applied in clinical practice; thus tests under extant copyright were excluded. The tests were selected based on Antinori et al. [8] recommended list for evaluating impairments in various ability domains. These tests measure performance in 7 cognitive domains, namely: Visual Learning Memory (Rey Osterrieth Complex Figure); Verbal Learning Memory (Story Recall); Language (Category Fluency); Motor speed (Grooved Pegboard Test); Speed of Information Processing (Trail Making Test A, Symbol Digit Modalities Test, Stroop Test); Attention and Working Memory (Digit Span Test); and Executive Functioning (Trail Making Tests B and C, Clock Drawing Test, Stroop incongruent condition). Commonly used bedside screeners like MMSE, MoCA and International HIV Dementia Scale (IHDS), which cover a range of neuropsychological abilities, were also administered. All tests were conducted in English. Normative data stratified by age and gender for this same battery was obtained from 258 HIV-negative individuals recruited from a pool of regular blood donors (refer to Appendix). The performance of the HIV-positive group was matched against demographic norms stratified by age and sex (stratification did not include educational level because everyone in the normative group had post-elementary education). Cognitive impairment was defined based on 2007 Frascati criteria [8] but excluding the criterion of level of functional impairment. Hence, cut-off scores on the MoCA and IHDS were not used singularly for determining cognitive impairment.

Data collected was entered and analysed using SPSS (PASW Statistics 18, SPSS, Inc., and Chicago, IL). The level of statistical significance was set at 0.05 for all tests. Patients were subsequently grouped into 2 cohorts, those classified with HAND by the comprehensive neuropsychological protocol, and those who were not.

The authors determined a priori that a battery consisting of 3 tests would be acceptable in our local setting considering the resources and time available for consultation and evaluation. All possible combinations of 3 cognitive tests from the comprehensive protocol were initially considered. The authors later decided to limit to 3-test combinations including MoCA as a requisite. Justifications for this decision include: firstly, MoCA is commonly used in Singapore to screen for cognitive impairment of any aetiology, and has been shown to be superior to MMSE [24, 25]. Secondly, it is easily adaptable to other languages and cultural settings [26] and has already been translated to 35 languages and dialects. Thirdly, multiple cognitive abilities (i.e., short-term memory recall, visuospatial ability, executive functioning, attention, working memory, language, orientation to time and place) can be assessed in a single tool, hence making MoCA a quick indicator of cognitive impairment due to any aetiology. This approach yielded 55 potential 3-test screening combinations.

To determine the best 3-test combination for the abbreviated screening test battery, we performed binary logistic regression analyses with presence of cognitive impairment as the dependent variable and combinations of the cognitive tests as independent variables. Based on predicted probabilities of a logistic regression model, each combination produced a propensity score. Sensitivity was plotted against specificity across varying cut-offs, generating a Receiver Operating Curve (ROC). Accordingly, each score corresponded to an ROC curve that illustrates the sensitivity and specificity of the combination for detecting cognitive impairment. The area under the curve (AUC), an index of effect size, was the primary result of the ROC analysis, and AUC summarizes the entire location of the ROC curve rather than depending on a specific operating point [27]. In short, AUC indicates the combined measurement of sensitivity and specificity of the relevant test. An area of 1.0 represents a perfect test and an area of 0.5 denotes an unfeasible test.

## Results

### Sample characteristics

Table 1 provides an overview of the demographic characteristics of HIV-positive individuals who presented to CDC for HIV care. Similarly, patients were classified according to presence of cognitive impairment, and the proportion is reflected in Table 1.

**Table 1:** Demographic characteristics and cognitive impairment classification of HIV patients who presented at CDC, N = 53

	Age, mean (SD)	Male	Years of education, mean (SD)	Ethnicity			
				Chinese	Malay	Indian	Others
Non-impaired (N = 25)	32.08 (9.50)	25 (100%)	14.08 (2.22)	12 (48.0%)	10 (40.0%)	2 (8.0%)	1 (4.0%)
Impaired (N = 28)	31.46 (8.42)	28 (100%)	13.68 (2.64)	22 (78.6%)	5 (17.9%)	1 (3.6%)	

	Marital status			Employment status	
	Single	Married	Divorced	Employed	Unemployed
Non-impaired (N = 25)	21 (84.0%)	3 (12.0%)	1 (4.0%)	16 (64.0%)	9 (36.0%)
Impaired (N = 28)	28 (100%)	–	–	23 (82.1%)	5 (17.9%)

	Alcohol consumption			Substance usage			Presence of non-HIV psychiatric conditions
	Nil	Stopped > 3 months	Still drinking	Nil	Stopped > 3 months	Still using	
Non-impaired (N = 25)	16 (64.0%)	2 (8.0%)	7 (28.0%)	21 (84.0%)	4 (16.0%)	–	2 (8.0%)
Impaired (N = 28)	16 (57.1%)	3 (10.7%)	8 (28.6%)	23 (82.1%)	4 (14.3%)	–	2 (7.1%)

All of the 53 patients recruited were male. Based on the scores of the cognitive screen, 52.8% of these patients were classified as impaired. Of the 28 patients who were classified with cognitive impairment, 21 (75%) scored above the cut-off values for MoCA (≥ 26) and IHDS (> 10). Unlike the findings of Chan et al. [2] no significant difference was found in the number of years of formal education between cognitively impaired and non-impaired patients. The relatively high impairment rate of 52.8% in this study sample was a result of stringent classification of HAND, unlike the classification applied in Chan et al.’s paper which probably underestimated the prevalence [2].

Table 2 presents the major HIV characteristics of the cognitively impaired and non-impaired HIV-positive patients.

**Table 2:** HIV characteristics of HIV patients based on cognitive impairment classification, N = 53

HIV characteristics	Non-impaired (N = 25)	Impaired (N = 28)
HIV duration (days)	261.84	369.82
CD4 Abs (< 200)	6 (24.0%)	5 (17.9%)
VL (% undetectable)	4 (16.0%)	5 (17.9%)
On HAARTs	21 (84.0%)	25 (89.3%)

### Sensitivity and specificity analyses

The 12 cognitive tests yielded 55 3-test combinations, with the inclusion of MoCA as one of the 3 tests. The top 15 combinations, ranked on the basis of highest test accuracy as measured by AUC, are presented in Table 3. Likewise, individual AUCs for MoCA and IHDS are reported in Table 3, and the scores demonstrate these tests are poor indicators when administered alone, affirming the use of a combined screening approach. The 15 combinations were then reviewed to identify those deemed easy and quick to administer at the bedside and during clinical consultations.

**Table 3:** MoCA, IHDS, and top 15 3-test combinations ranked according to the area under the curve (AUC) in Receiver Operating Characteristic analysis

Combination	Tests	AUC	95% CI
	MoCA alone	0.516	[0.355, 0.677]
	IHDS alone	0.572	[0.413, 0.731]
1	MoCA + SR + Stroop Word	0.857	[0.752, 0.962]
2	MoCA + CF + Stroop Word	0.854	[0.748, 0.960]
3	MoCA + SR + CF	0.836	[0.727, 0.945]
4	MoCA + ROCF + SR	0.832	[0.721, 0.944]
5	MoCA + SR + Stroop Name	0.815	[0.696, 0.934]
*6*	*MoCA* + *GPT* + *SR*	0.798	[0.678, 0.919]
7	MoCA + CF + Stroop Name	0.798	[0.675, 0.922]
8	MoCA + GPT + CF	0.791	[0.665, 0.917]
9	MoCA + CF + DS	0.791	[0.662, 0.920]
10	MoCA + TMT + SR	0.788	[0.666, 0.909]
11	MoCA + ROCF + CF	0.788	[0.657, 0.919]
*12*	*MoCA* + *SR* + *SDMT*	0.788	[0.665, 0.911]
13	MoCA + CF + CDT	0.788	[0.656, 0.919]
14	MoCA + IHDS + CF	0.787	[0.655, 0.919]
*15*	*MoCA* + *IHDS* + *SR*	0.778	[0.652, 0.905]

CI, confidence interval; SR, Story Recall; Stroop Word, Stroop Color-Word (Inhibition); CF, category fluency; ROCF, Rey Osterrieth Complex Figure; Stroop Name, Stroop Color Naming; GPT, Grooved Pegboard Test; DS, Digit Span; TMT, Trail Making Test; SDMT, Symbol Digit Modalities Test; CDT, Clock Drawing Task

Of the 15 combinations listed in Table 3, the first combination generated the highest AUC of 0.857, demonstrating good sensitivity and specificity as a screening battery. However, upon further examination of the top 15 combinations, the authors found that majority of the combinations may not be appropriate as they placed a strong emphasis on the ability to read. Patients whose dominant language is not English or who have weaker language abilities might experience difficulty in comprehending the Stroop Test. The ROCF was found to be more difficult and time-consuming to score by the authors, making combination 4 a less preferred option. Combinations 3 and 10 were also removed from consideration as MoCA already includes items which measure category fluency and executive functioning respectively. After deliberation and discussion, the authors agreed on combinations 6, 12 and 15 to recommend as options for a brief HAND screening battery (see italic combinations in Table 3). These combinations were found to classify HIV patients on cognitive impairment with acceptable accuracy, as denoted by their AUC score of above 0.7.

## Discussion

With the objective of improving HIV patient management, cognitive assessments at bedside or during clinic consultations are necessary in detecting HIV patients with HAND for possible early intervention. An effective screening battery for detecting cognitive impairment should be concise, easy to administer and both specific and sensitive. To satisfy these demands, the screening battery should combine the fewest sensitive neuropsychological tools to yield the highest possible sensitivity and specificity. This is imperative given that impaired cognition can negatively impact medication adherence, employment and compromise quality of life [3, 4]. Early detection would allow interventions to slow or potentially reverse the progression of HAND.

As alternatives to using the complete neurocognitive diagnostic protocol or stand-alone diagnostic tests for HAND, the authors have identified 3 brief screening batteries. These 3-test combinations were found to detect cognitive impairment in HIV patients with a fairly high degree of accuracy, based on the gold standard of a larger comprehensive neurocognitive protocol. The recommended tests are recognised for measuring neurocognitive decline and are deemed suitable for implementation in resource-limited settings in many Asian countries where English is not the native language, since the tests have either been translated and validated in Asian languages [28, 29], or do not require the ability to read in English.

In addition to the cognitive functions tested for in MoCA, screening batteries 6, 12 and 15 assess psychomotor functioning, verbal learning memory and information processing speed, which are among the commonly impaired neurocognitive abilities in HIV-infected patients [8, 13]. Congruent with previous findings, tests of verbal learning, processing speed and psychomotor coordination offer the best combinations in detecting HAND [13], yet even specified subset scores of the MOCA yielded insufficient accuracy [30]. This result underlines the fact that impairments in the domains of motor speed, language and executive functioning are common among patients with HAND but require more specific tools for detection [1–4, 30]. It was previously shown that both cortical and subcortical functions are frequently affected in South Asian HIV patients, therefore the application of a comprehensive screening battery would aid in detecting multi-domain cognitive impairment [2]. The remaining batteries were excluded due to their stronger emphasis on language proficiency. We believe these screening batteries can be influenced by level of education and literacy; hence if they were used, the recommended cut-off values would have to be stratified by educational levels. Subsequently, the existence of several spoken languages and dialects in multi-ethnic countries like Singapore further complicates the use of these cognitive screening tools for the early diagnosis of HAND.

A list of options is presented in the current paper, rather than endorsing a specific battery. While it may be argued this can give rise to inconsistency in administration, we think the options allow for greater flexibility in conducting cognitive screenings in various clinical settings. If sensitivity and specificity were the most important criteria, the top ranked combination 6, would seem to be most reasonable choice. However, if interviewers find themselves unable to secure the grooved pegboard for GPT, they can consider switching to combinations 12 or 15 which comprise only of paper-and-pen tests. Likewise, the set of options would prove to be advantageous if multiple screenings were to be conducted over time. Cognitive performance can vary periodically and neuropsychological tests are susceptible to practice effects [8]. Interviewers could consider administering the 3 combinations interchangeably at different assessment time points to minimize possible bias or contamination through learning or practice effect.

The existing nosology denotes ANI as a distinct category from MND and HAD. Given the increasing accessibility of HAART and hence reduced morbidity from HIV/AIDS, including neurocognitive impairment in HIV-infected populations, [1, 5] the use of a single neurocognitive test to screen for all severities of HAND might not suffice. Most previous efforts have emerged from studies in Western populations, and there is a paucity of evidence for a screening battery for detecting HAND in Asian patients. Several studies have examined the utility of MoCA in screening HAND, but have questioned the validity of utilizing it singularly [17, 24, 26, 31, 32]. More recently, Kim et al. performed similar screening HAND selection in a South Korean population [30]. Their findings demonstrated increased predictive power when TMT-A is used in conjunction with MoCA. On the same note, a study conducted in Thailand by Chalermchai et al. [22] found high rates of milder forms of HAND and its associated deleterious effect on quality of life. Although efficient and commonly utilised, IHDS was found to perform less favorably in identifying milder forms of HAND in this setting. This study demonstrated improvement in sensitivity of the HAND screening battery with the addition of Trail Making Test A, a test of psychomotor speed. The administration and interpretation of the screening combinations recommended here are straightforward and require minimal training, and can be applied in various international settings.

Our study has several limitations. Neither information nor performance on patients’ functional impairment was measured, limiting the classification of HAND in our sample. The sample size is small, thus possibly limiting the ability to test the accuracy of the screening combinations. However, the total number of reported HIV diagnoses in Singapore totaled 7982 as of end 2017, with an incidence of 0.109, [33] limiting the recruitment of large samples for the current study. Of which, 243 newly-diagnosed patients were presented to CDC during the period of study and recruitment rate approximates 21% of this total cohort. The majority of HIV-positive individuals receive treatment at the CDC, thus the current sample likely approximates the true prevalence of HAND in Singapore. Secondly, the screening combinations recommended in this paper are derived from a sample of well-educated young, male HIV-infected individuals. Our sample consisted only of males—reflecting the epidemiology of HIV infection in Singapore, where over 90% of HIV-infected individuals are male. The current sample may hence have been unable to account for potential gender differences in cognition. Therefore, findings may only be generalizable to HIV-infected populations with similar characteristics as that of our study, and we propose these combinations to be the most suitable in demographically comparable Asian populations. Future studies with larger multi-ethnic sample sizes are hence needed. Importantly, validation of the recommended batteries in HIV-positive samples that are less gender-biased and across varied educational backgrounds and spoken languages can now be realistically executed.

Even though we excluded individuals with overt cognitive impairment and clinically significant anxiety and depression from our sample, we did not specifically exclude those with comorbid neurological conditions. Considering the high level of comorbidity within the general HIV population and multifactorial aetiologies of cognitive impairment, clinical judgment is still required to ascertain the diagnosis of HAND. Hence, further studies could also consider the capacity of the current recommended battery combinations to differentiate between HAND and other dementing illnesses such as Alzheimer’s disease, vascular dementia or frontotemporal dementia.

In summary, we propose combinations of widely-accepted neuropsychological tests with short implementation time that demonstrate adequate sensitivity and specificity as compared with a more time-intensive neuropsychological test battery. These could prove to be valuable in initial diagnosis of HAND in time and human resource-limited non-English-predominant clinical settings. Practitioners or members of the medical team can select the most appropriate screening battery depending on the setting and testing resources available. To determine specific cut-off scores or even a global cognitive score for these recommended batteries, further validation and demographically corrected normative data in larger samples are necessary. In the absence of a clear diagnostic gold standard, the best screening paradigm is difficult to define. This indicates a need for continued research to better determine diagnostic categories and procedures of HAND. With more robust prevalence data, our recommended screening batteries can then be more stringently accessed and validated. Nonetheless, with the recommended screening batteries, we intend to raise the awareness of screening for HAND among clinicians, particularly in Southeast Asia, where it represents a significant untreated burden and where more needs to be done to reduce the negative impact of neurocognitive decline on patients’ quality of life.

## Conclusions

Previously, there were no locally validated tools for the screening and detection of cognitive impairment in the HIV population in Singapore. This paper proposes 3 brief, conventional neurocognitive screening battery combinations that can be administered flexibly in various settings.

## Appendix

See Tables 4 and 5.

**Table 4:** Demographic characteristics of 258 HIV-negative regular blood donors

Characteristics	Male (N = 105)	Female (N = 153)
Age, mean (SD)	37.59 (11.0)	42.33 (10.23)
Ethnicity		
Chinese	73 (69.5%)	130 (85.0%)
Malay	10 (9.5%)	6 (3.9%)
Indian	14 (13.3%)	11 (7.2%)
Others	8 (7.6%)	6 (3.9%)
Level of education		
Secondary	8 (7.6%)	37 (24.2%)
Tertiary	80 (76.2%)	100 (65.4%)
Post-tertiary	17 (16.2%)	16 (10.5%)
Employment status		
Employed	87 (82.9%)	120 (78.4%)
Unemployed	18 (17.1%)	33 (21.6%)

**Table 5:** Normative cognitive performance of 258 HIV-negative regular blood donors

	Male		Female	
	21–40 years old (N = 60)	41–66 years old (N = 45)	21–40 years old (N = 66)	41–66 years old (N = 87)
ROCF immediate	29.9 (2.5)	30.3 (2.2)	29.9 (1.8)	30.3 (2.7)
ROCF delayed	17.8 (4.9)	17.9 (6.6)	17.7 (4.9)	15.7 (6.1)
SR immediate	9.8 (2.5)	9.6 (2.6)	11.0 (2.2)	9.7 (2.2)
SR delayed	9.7 (2.3)	9.0 (2.6)	10.3 (2.3)	9.0 (2.2)
TMT (average time taken/s)	41.1 (11.6)	49.2 (10.3)	40.5 (10.7)	46.8 (12.9)
CF	20.0 (4.3)	18.1 (4.8)	19.4 (4.7)	19.1 (4.9)
DS forward	9.8 (1.8)	9.5 (1.8)	9.8 (1.8)	9.5 (2.0)
DS backward	7.3 (2.6)	6.8 (1.9)	7.0 (2.3)	7.3 (2.4)
CDT	4.5 (0.6)	4.4 (0.7)	4.6 (0.6)	4.6 (0.6)
GPT dominant hand (time taken/s)	64.5 (11.1)	68.3 (11.3)	61.3 (7.5)	67.3 (10.7)
GPT non-dominant hand (time taken/s)	70.8 (10.9)	74.8 (12.3)	68.5 (8.9)	73.9 (11.6)
SDMT	59.4 (9.9)	53.0 (8.0)	60.6 (9.3)	55.4 (10.8)
Stroop word	99.2 (15.7)	98.4 (17.0)	98.4 (14.4)	95.9 (14.9)
Stroop name	71.1 (11.8)	70.1 (10.9)	71.9 (11.1)	68.5 (11.3)
Stroop test (word-name)	42.7 (8.8)	36.7 (9.5)	42.8 (7.7)	38.9 (9.5)
MoCA	> 25.0	> 25.0	> 25.0	> 25.0
MMSE	> 24.0	> 24.0	> 24.0	> 24.0
IHDS	> 10.0	> 10.0	> 10.0	> 10.0

ROCF, Rey Osterrieth Complex Figure; SR, Story Recall; TMT, Trail Making Test; CF, Category Fluency; DS, Digit Span; CDT, Clock Drawing Task; GPT, Grooved Pegboard Test; SDMT, Symbol Digit Modalities Test; Stroop Word, Stroop Color-Word (Inhibition); Stroop Name, Stroop Color Naming
